# The Operations of Surgery—A Systematic Hand Book for Practitioners, Students and Surgeons

**Published:** 1889-05

**Authors:** 


**The Operations of Surgery: A Systematic Hand-Book for Practitioners, Students, and Surgeons.** By W. H. A. Jacobson, F. R. C. S., Assistant Surgeon Guy's Hospital; Teacher of Operative Surgery, and Joint Teacher of Practical Surgery in the Medical School; Surgeon to the Royal Hospital for Children and Women. With 199 illustrations. Philadelphia: P. Blakiston, Son & Co., No. 1012 Walnut street. 1889. Buffalo: Peter Paul & Bro. Price, $5.00.

Since the appearance, in 1879, of Dr. Stephen Smith's Operative Surgery, no work of a similar character has been given to the profession. Smith's Surgery has, by many, been thought to be too concise, and, although full of meat, and of nothing but meat, is a difficult work to read. Smith's book is eminently a work on operative surgery, leaving out almost everything about indications and prognosis, and particularly paying attention to the various details of the different operations.

Jacobson's work, on the other hand, strikes the writer more as a treatise on special surgery, discusses the indications and contra-indications of the various operations fully, pays due attention to prognosis and the dangers of the operations, the causes of failure, etc. It is, therefore, much easier and pleasanter reading. The work contains six parts, treating of Operations on the Upper Extremity, on the Head and Neck, on the Thorax, on the Abdomen, on the Lower Extremity, and a very short chapter on Operations on the Vertebral Canal.

As a general rule, it brings our knowledge of these subjects up to date, and yet we miss several modern operations, particularly from the continent, while everything English is scrupulously mentioned, and English authors often given credit for improvements which belong to continental surgeons. While Horsley's brilliant operations on the brain are fully and interestingly treated, and the more modern abdominal operations, as Loreta's dilatation of the pylorus, extirpation of the cancerous stomach, extirpation of the larynx, are fully treated, we miss other operations, perhaps less brilliant in their conception, but, nevertheless, more frequently resorted to. The operation of arthrectomy, which has revolutionized, on the continent, the treatment of tuberculous joints, is not mentioned at all, although here and there due importance is given to the removal of all tuberculous tissue. Schede's blood-clot method is not mentioned at all; neither is Thiersch's skin-grafting, an operation of much greater benefit to suffering humanity than the pylorectomy. Estlander's name is scarcely mentioned, nor is Schede's modification of his operation. Intubation of the larynx, and perityphlitis and its operations are completely left out.

It is, of course, easy to find fault, but, taken as a whole, the book will be received with gratitude by the profession, and find a place in every active surgeon's revolving book-case.

